# Supplementary material for: Effects of Whey Protein Supplementation on Aortic Stiffness, Cerebral Blood Flow, and Cognitive Function in Community-Dwelling Older Adults: Findings from the ANCHORS A-WHEY Clinical Trial
Source: Nutrients. 2020 Apr 10;12(4):1054. doi: 10.3390/nu12041054 (PMC7230701; doi:10.3390/nu12041054)
Supplement: Supplementary file 1 [file nutrients-12-01054-s001.pdf]

Table S1. Memory, impulsivity, emotion identification, and response speed at baseline and 12-wk in WPI and CHO groups (mean±SD).

Task	Construct	WPI Baseline	WPI 12-wk	CHO Baseline	CHO 12-wk	Effects, p-value (z/sqrt[n]) Group	Time	GxT	df
**Memory**									
Immediate	Recognition (#)	56±4	57±4	56±4	56±4	0.99(0.09)	0.34(0.16)	0.99(0.04)	96
	Learning Rate	0.62±0.98	0.36±0.91	0.72±0.90	0.37±0.72	0.99(0.05)	0.01(0.03)	0.99(0.1)	96
	Intrusion Errors (#)	3±4	3±4	4±4	3±4	0.99(0.09)	0.34(0.16)	0.99(0.04)	96
Delayed	Recognition	19±2	19±1	19±1	19±2	0.99(0.08)	0.99(0.04)	0.21(0.19)	94
	Intrusion Errors (#)	0.88±1.60	0.58±0.99	0.89±1.40	1.09±1.67	0.99(0.08)	0.99(0.04)	0.21(0.19)	94
**Impulsivity**									
Go/No-Go	Speed (ms)	352.2±58.0	357.9±56.2	362.4±47.2	360.0±49.6	0.93(0.11)	0.93(0.1)	0.36(0.16)	92
	Commission Errors (#)	6±8	4±3	5±6	4±3	0.99(0.09)	0.04(0.25)	0.75(0.12)	92
	Omission Errors (#)	2±3	1±1	2±2	2±3	0.99(0.05)	0.01(0.3)	0.46(0.15)	92
**Emotion Identification**									
Immediate	Correct (%)	71.3±7.5	73.9±7.2	70.2±9.1	70.5±9.6	0.99(0.1)	0.1(0.22)	0.56(0.13)	96
	RT (ms)	3179.2±733.3	2944.9±832.9*	3066.1±1038.6	3063.1±1104.0	0.99(0.09)	0(0.34)	**0.01(0.29)**	96
Delayed	Correct (%)	97.8±3.4	99.4±1.7	97.1±4.1	97.8±3.9	0.6(0.16)	0.01(0.29)	0.99(0.06)	96
	RT (ms)	1687.6±316.1	1460.9±252.1	1718.4±422.2	1571.5±369.6	0.99(0.08)	0(0.66)	0.44(0.15)	96
**Response Speed**									
Motor Tapping	Speed (#)	165±23	166±24	167±26	168±25	0.99(0.01)	0.9(0.11)	0.99(0.01)	89

All data analyzed using non-parametric analyses with Bonferroni correction. Effect sizes calculated as Z/√n. WPI, whey protein isolate; CHO, carbohydrate; RT, reaction time. *p<0.05 vs Baseline.

Table S2. Working memory, and attention and concentration at baseline and 12-wk in Whey and Carbohydrate groups (mean±SD).

Task	Construct	WPI Baseline	WPI 12-wk	CHO Baseline	CHO 12-wk	Effects, p-value (z/sqrt[n]) Group	Time	GxT	df
**Working Memory Capacity**									
Digit Span (Forward)	Recall Span (#)	7±1	7±1	6±2	6±1	0.60(0.15)	0.99(0.08)	0.99(0.05)	87
	Correct (#)	8±2	8±2	7±3	8±2	0.85(0.13)	0.95(0.11)	0.99(0.08)	87
**Attention and Concentration**									
Continuous Performance Test	RT (ms)	549.8±136.1	558.1±107.5	577.2±163.8	544.7±114.4	0.99(0.07)	0.99(0)	0.22(0.18)	95
	Commission Errors (#)	4±8	2±6	1±1	2±7	0.99(0.08)	0.16(0.2)	0.99(0.03)	95
	Omission Errors (#)	1±1	1±1	1±2	1±1	0.99(0.04)	0.99(0.08)	0.72(0.12)	98

All data analyzed using non-parametric analyses with Bonferroni correction. Effect sizes calculated as Z/√n. WPI, whey protein isolate; CHO, carbohydrate; RT, reaction time.

Table S3. Information processing efficiency and executive function at baseline and 12-wk in Whey and Carbohydrate groups (mean±SD).

Task	Construct	WPI Baseline	WPI 12-wk	CHO Baseline	CHO 12-wk	Effects, p-value (z/sqrt[n]) Group	Time	GxT	df
**Information Processing Efficiency**									
Switching of Attention	Duration (s)	64.43±18.23	58.05±17.40	66.10±20.09	59.01±17.29	0.99(0.03)	**0.001 (0.55)**	0.99(0.01)	94
	Connection Time (ms)	2531±762	2315±762	2727±1210	2384±766	0.99(0.05)	**0.001(0.49)**	0.99(0.04)	94
	Accuracy (%)	0.73±1.72	0.51±1.35	0.58±1.23	0.11±0.32	0.99(0.07)	0.07(0.23)	0.90(0.11)	94
Choice RT	RT (ms)	423.7±108.2	404.0±58.5	440.4±120.4	469.8±161.4	0.49(0.17)	0.99(0.02)	0.31(0.17)	89
Verbal Interference (Word)	Correct (#)	11±4	11±4	11±4	11±4	0.99(0.04)	0.99(0.07)	0.99(0.01)	93
	Errors (#)	0.3±0.8	0.3±0.7	0.2±0.6	0.4±1.2	0.99(0.08)	0.99(0.02)	0.99(0.02)	93
	RT (ms)	1179.6±615.2	1918.3±954.8	1999.3±1597.6	1792.7±753.9	0.99(0.04)	0.99(0.06)	0.99(0.09)	93
Verbal Interference (Color)	Correct (#)	8.5±3.0	9.8±3.1	7.9±3.6	9.3±3.5	0.99(0.07)	**0.001 (0.47)**	0.99(0.00)	95
	Errors (#)	0.3±0.8	0.3±0.7	0.2±0.6	0.4±1.2	0.99(0.08)	0.99(0.02)	0.99(0.02)	93
	RT (ms)	1779.6±615.2	1918.3±954.8	1999.3±1957.6	1792.7±753.9	0.99(0.04)	0.99(0.06)	0.99(0.09)	93
**Executive Function**									
Maze	Trials (#)	9.5±3.5	9.6±3.7	9.8±3.0	8.8±3.5	0.99(0.09)	0.88(0.11)	0.37(0.16)	93
	Completion Time (s)	345.05±144.78	295.45±114.32	326.23±99.74	281.25±113.18	0.99(0.04)	**0.001 (0.52)**	0.99(0.02)	93
	Learning time (s)	299.89±126.76	255.41±100.09	290.17±89.89	244.61±109.38	0.99(0.07)	**0.001 (0.49)**	0.99(0.00)	93
	Accuracy (%)	49.2±31.9	39.7±26.1	51.6±26.6	40.8±32.8	0.99(0.05)	**0.001 (0.5)**	0.93(0.1)	93
	Overruns (#)	20±17	16±12	23±17	18±19	0.99(0.06)	**0.001 (0.34)**	0.99(0.06)	93

All data analyzed using non-parametric analyses with Bonferroni correction. Effect sizes calculated as Z/√n. WPI, whey protein isolate; CHO, carbohydrate; RT, reaction time.
